# L-sign in appendicitis: a case series

**DOI:** 10.4076/1757-1626-2-8117

**Published:** 2009-08-10

**Authors:** Imtiaz Wani

**Affiliations:** Department of General Surgery/Accident and Emergency, Sheri-Kashmir Instuite of Medical SciencesSrinagar, KashmirIndia

## Abstract

**Introduction:**

Clinical signs have been always of significant value in the diagnosis of appendicitis. Specific clinical signs are elicited coupled with routine laboratory and radiological investigations in making the diagnosis of acute appendicitis. L-sign in appendicitis is similar to the well known Rovsing’s sign and is based on the same principle but is elicited on right side on the lateral abdominal wall.

**Case presentation:**

A case series of 3 patients is presented here who had the L-sign in acute appendicitis. All had Rovsing’s sign positive and the histopathological evidence of appendicitis.

**Conclusion:**

L-sign in appendicitis is similar to the Rovsing’s sign in diagnosis of appendicitis but elicited on right side.

## Method of eliciting L-sign

An index finger tap given on lateral abdomen wall at L-point. Starting from upper flank in a line which is perpendicular to the line passing through highest point of iliac crest, an index or middle finger tap is given on the lateral abdomen wall till the L-point reached, patients complains of localized tenderness in right iliac fossa, more than at the L-point and this localized tenderness felt in right iliac fossa after applying tap on L-point is called as L-sign.

## Case presentations

### Case report 1

12-year-old Kashmiri boy of Indian ethnicity had right lower abdominal pain, fever and nausea of 23 hours’ duration. General physical examination revealed pulse of 98/min, blood pressure of 100/70 mm Hg and fever. Per abdominal examination findings were presence of tenderness in right lower quadrant, rebound tenderness, Rovsing’s sign and L-sign. In this case, L-point was adjacent to highest point of iliac crest on lateral abdominal wall. X-ray abdomen was unremarkable. On ultrasonography of abdomen, probe tenderness was recorded. There was presence of leucocytosis of 9, 5000/mm^3^. Patient had appendectomy and 12.6 centimeter inflamed appendix preileal in position with fecaliths inside was seen. Histopathology of specimen revealed appendicitis. Patient regularly attended our follow up clinics and was uneventful.

### Case report 2

A 13-year-old Kashmiri girl of Indian ethnicity presented with pain right lower abdomen, nausea and a vomiting of 11 hours duration. Initially she had epigastria pain and then had shift of pain to the right lower abdomen. There was a history of anorexia. General physical examination revealed low grade fever, pulse of 96/min and blood pressure of 110/80 mm Hg. Systemic examination was normal. Positive findings recorded on abdominal examination were deep tenderness in right iliac fossa and Rovsing’s sign. An L-sign was elicited in the same way as in case 1and was found to be positive at the same point as in case 1. Leucocytosis of 12,400/mm^3^ with with neutrophilia of 80%. Localised ileus was present on X-ray abdomen in right iliac area. Ultrasongraphy abdomen finding was a non-compressible tube like structure suggestive of appendicitis. Patient had appendectomy and an inflamed appendix measuring 10.3 centimeter long which was retrocaecal in position was seen. Histopathology was consistent with acute appendicitis. Postoperative period was uneventful.

### Case report 3

An 18-year-old Kashmiri male of Indian ethnicity had right lower abdominal pain, vomiting, loose motion with fever of 2 days duration. On general physical examination tachycardia of 93/min and mild fever was recorded. On per abdominal examination there was rebound tenderness in right iliac area. Rovsing’s sign was positive. An L-sign was elicited in the same way as in case 1and case 2 and was found to be positive but this point being slightly higher about 1.5 centimeters above the highest point of iliac crest X-ray abdomen showed localized ileus in right iliac area. Ultrasonography abdomen revealed probe tenderness in right lower abdomen. A diagnosis of appendicitis was made and had appendectomy. Peroperative findings revealed a caecum slightly high up in position, inflamed appendix being post ileal in position with pus inside. Histopathology was consistent with acute appendicitis Follow up period was normal.

## Discussion

Appendicitis is a common surgical emergency. Diagnosis of appendicitis is based primarily on the patient’s history and the physical examination and these two are still the most important basis for decision making for the diagnosis of appendicitis. Diagnostic accuracy achieved by history and physical examination had remained at about 80 percent [1].

Physical signs resulting from various maneuvers designed to elicit peritoneal pain can be helpful in the diagnosis [2]. Clinical signs persists as the most sensitive diagnostic method having 93.9%, so these remains the cornerstone of management [3,4]. If the diagnosis of acute appendicitis is clear from the history and the physical examination, no further testing is needed. A clinician has to develop keen training in eliciting correctly signs for diagnosis of appendicitis. The various specific clinical signs in right iliac fossa indicate localized peritonitis. A lack of standardization of clinical signs accounts the low interobserver reliability of clinical findings [5].

L-sign can be simply elicited by percussing at a point adjacent to highest point of iliac crest area on the lateral abdominal wall. Percussion started from upper flank in the line which is called as L-line being perpendicular to the line which passes through highest point of iliac crest, the L-point is reached where this sign was elicited and the patient had localized tenderness right iliac fossa. This L-point and Mc Burney’s point join each other at right angles on abdominal wall. The effort of eliciting “L Sign” simulates signs for eliciting localized peritonitis and this sign is based on eliciting pain in base region of inflamed appendix. In this sign, transmitted pulsation or vibration irritating the base or lining of the peritoneum at the place where the anterior peritoneum comes is in contact with the inflamed viscus. Displacement of contents or air in caecum by stimulating L-point irritating the base of appendix could be another possible mechanism. The mechanism for L-sign simulates as that for Rovsing’s sign that a momentary vibration or displacement of the abdominal wall or abdominal contents initiated by pressure on left iliac fossa and passing across right iliac fossa and causing friction between the inflamed viscus and the overlying anterior peritoneum with resulting pain [6,7]. Conditions other than appendicitis in which L-sign can be found are mesentric adenitis in children torsion or the rupture of right ovarian cyst, pelvic inflammatory disease, Meckel’ diverticulitis and symptomatic lower ureteric stone etc. The L-sign in appendicitis sign can be more appropriately called as “Right Rovsing’s sign.”
